# Chronic Ulcer of the Legs

**Published:** 1892-05-07

**Authors:** 


### CHRONIC ULCERS OF THE LEGS.

There have been great advances made of late not only in the pathology of these ulcers but also in our knowledge of what must be aimed at in treatment. The value of antisepsis, of pressure, of strapping and elastic bandages, of elevation and skin grafting is acknowledged, but there is the greatest diversity in their application. It is instructive to compare the methods employed in the various Paris hospitals, as described in *La Semaine Medicale.* Thus Professor Verneuil employs a 2 per cent. carbolic lotion, and afterwards aseptic dressings, with rest and elevation of the limb. Dupluy purifies them in the same way, but in atonic ulcers uses chlorine water, changing the dressing every few hours. When granulations appear, a thick aseptic pad is bandaged on so as to exert a gentle pressure, and changed only once a week. For some of these atonic ulcers storax is used as a stimulant; and for inflammatory ones, compresses made with spirits of camphor are employed. Large surfaces are grafted after Thiersch's or Reverdin's methods. Le Dentu, on the other hand, thinks that varicose ulcers succeed best with a 2 per cent. chloride of calcium dressing, and in some cases gets good results by dusting with sesquioxide of iron. Dr. Desprez contends that ulcers on the shins arise either in connection with a broken-down varicose vein, or from a patch of varicose erythema. In first attacks, at least, he uses strapping, applied in Baynton's manner, surrounded by an elastic bandage, and renewed every four days. Healing is brought about much quicker if the patient remains in bed. In relapses he reduces the inflammation by means of a poultice, and then begins again his strappings. After complete healing, he keeps the skin covered by a layer of powder and an elastic bandage outside it.

Dr. Berger applies a solution of sublimate until there are good granulations, then storax dressings. After three or four days he returns to the sublimate bandages, then again the storax, and so on alternately until the margins are formed of new skin. Next, by Reverdin's method of grafting, he breaks up the large area of the ulcer into many smaller ones, which heal over with comparative ease. During the whole treatment we must insist on complete rest in bed until some weeks after the wound is completely healed over.

In ulcers with hard thick edges, and where he finds ecchymotic colouring of the base of the ulcer, he uses Dolbeau's circular incision with good results. This is made at two centimetres from the margin of the ulcer, through the entire thickness of the skin with the superficial veins. Severe bleeding will be easily stopped by compression and elevation of the limb. The immediate result of the incision is usually seen in the fading of the extreme redness of the surface, and, later on, in a remarkably quickened tendency to heal. One can, as a rule, do without skin transplantation if Dolbeau's incision is carried out thoroughly.

Dr. Terrier usually obtains quick healing in small varicose ulcers by a single antiseptic dressing, and rest in bed. In larger ones he employs Reverdin's or Thiersch's plan of grafting.

According to Quénu, we have the three following indications to consider in the treatment of varicose ulcers of the shins:—(1) Antisepsis of the wound to get rid of complications; (2) compression of the wound and its neighbourhood to get rid of the œdema and venous stasis; (3) stimulation of the atonic cellular elements. These aims are attained, he thinks, by boric acid dressings, and later on, after careful purification of the wound, by the use of a dressing containing one per cent. solution of sulphate of copper. This dressing is compressed by an elastic bandage, and renewed every three days. In a few cases a weaker solution is used, or the dressing is only applied for a few hours daily.

Reclus considers we should disinfect these wounds twice a day carefully with warm Van Swieten's solution, as well as raise the limb and bandage from the toe to the middle of the thigh.

Exuberant granulations should be repressed by the thermo-cautery, which is also recommended for ulcers with callous edges. Transplantation of skin is also used for the larger specimens.

In ulcers of small extent and depth and slightly thickened edges, the five per cent. copper solution is recommended by Schwartz. This is applied twice a day, and later on is replaced by strapping, while in callous ulcers Dolbeau's incision, scarification, and transplantation may be needed. Occasionally, even amputation may be desirable in very exceptional cases. Labbé prefers for dressings one of ten per cent. chlorine water, and later on strapping. In obstinate cases he has found the Dolbeau's incisions are most valuable.

Very similar is Gunther's plan. He first sterilizes the ulcer, and then powders it with salol and talc in equal parts, and at the same time applies massage to the surrounding parts. After complications are thus got rid of, he uses strapping to complete the cure.

There is a common idea running through these various methods. They all recognise the need of getting rid of microbes, and of indurated margins, and they all aim at restoring the venous, lymphatic, and arterial circulations, which are more or less interfered with. For an ulcer fails to heal just so long as impediments to the circulation or microbes overcome the normal cell growth. When these are removed the parts quickly regain their usual contour. The use of large skin grafts notably hastens the cure of some cases. Dr. McBurney has modified Thiersch's method; he washes the ulcers and the skin to be transplanted with sublimate lotion, and next with common salt in sterilized water (6 in 1,000). The surface of the granulations and the callous edges are curetted or shaved off after an Esmarch's bandage has been applied to the limb. Thin slices of skin, four or five inches long and an inch wide, are pared off another part of the body by a keen razor and placed smoothly and accurately on the floor of the ulcer. They are covered by strips of protective moistened in the salt solution, and over these a thick moist dressing is packed.

After all some of the most troublesome ulcers to treat are small, healthy-looking ones, which seem always to be on the point of healing. It often becomes with them a very difficult question whether to persevere with ordinary dressings or to remove the entire surface of the ulcer, and to graft on an entire new layer by the above-mentioned method. Painful ulcers, too, often tax our resources to manage. We may dust with antipyrin, aristol, or dress with nitrate of silver or carbolic, or employ poultices, continuous baths, or anodyne collodion, but in obstinate cases curetting the surface or Dolbeau's incision may have to be employed.

